# S3-4 Social prescription: a meaning for general medicine in Belgium? A qualitative exploration with patients and healthcare providers about the physical literacy counselling program

**DOI:** 10.1093/eurpub/ckad133.017

**Published:** 2023-09-11

**Authors:** Géraldine Godin, Alexandre Mouton

**Affiliations:** Université Catholique de Louvain, Belgium; Sport Sciences Department, University of Liege, Belgium

## Abstract

**Purpose:**

In a society where the population is aging and chronic diseases are more and more present, social determinants of health occupy a growing place in primary care medicine. As the demand for holistic care is growing, the bio-psycho-social model takes on its full meaning within primary health care.(1) The “social prescribing” model, introduced in many countries in recent years, allows the integration of non-medical care into the primary health care system without overburdening the health professional. (2) Social prescribing enables general practitioners and others primary care professionals to refer people to a range of non clinical services to support their health such as the intervention of physical activity specialists.

**Methods:**

This study defines social prescribing and discusses successful aspects and challenges of the model in targeted prescription of physical activity. It also evaluates a pilot project set up in Belgium in 2022 to test the model. The pilot project aimed at promoting, through doctors and physiotherapists of the medical center, the orientation of patients to physical literacy counselling meetings leaded by a specialized physical educator. The purpose of this study was then to explore, qualitatively, patients and staff of a medical center about their opinion over a physical literacy counselling program. Interviews of patients and healthcare providers were undertaken during this project.

**Results:**

The strengths of the pilot project developed include successful implementation of holistic patient-centered care, strengthening of patients’ mental and physical well-being alongside empowering their health. In addition, the project successfully foster preventive care and demonstrates how primary care may transform into primary health care. However, many challenges remain including the sustainability of the model itself, the availability of the services offered, the financial support and the low commitment of doctors and patients.

**Conclusions:**

this study highlights first guidelines to introduce the model into the practice of primary healthcare in Belgium. Although the project shows limitations, the theoretical model remains promising and opens up new potential horizons for the practice of general medicine in Belgium.

**Funding Source:**

This study received no external funding.

